# Nonlinear Dynamic Modeling and Analysis of an L-Shaped Multi-Beam Jointed Structure with Tip Mass

**DOI:** 10.3390/ma14237279

**Published:** 2021-11-28

**Authors:** Jin Wei, Tao Yu, Dongping Jin, Mei Liu, Dengqing Cao, Jinjie Wang

**Affiliations:** 1 School of Electromechanical and Automotive Engineering, Yantai University, Yantai 264005, China; yt_126@126.com (T.Y.); wangjinjie@ytu.edu.cn (J.W.); 2 State Key Laboratory of Mechanics and Control of Mechanical Structures, Nanjing University of Aeronautics and Astronautics, Nanjing 210016, China; jindp@nuaa.edu.cn; 3 School of Astronautics, Harbin Institute of Technology, Harbin 150001, China; liumeip@163.com (M.L.); dqcao@hit.edu.cn (D.C.)

**Keywords:** L-shaped multi-beam jointed structure, beams nonlinearities, joints nonlinearities, nonlinear dynamic model, nonlinear dynamic characteristic

## Abstract

A dynamic model of an L-shaped multi-beam joint structure is presented to investigate the nonlinear dynamic behavior of the system. Firstly, the nonlinear partial differential equations (PDEs) of motion for the beams, the governing equations of the tip mass, and their matching conditions and boundary conditions are obtained. The natural frequencies and the global mode shapes of the linearized model of the system are determined, and the orthogonality relations of the global mode shapes are established. Then, the global mode shapes and their orthogonality relations are used to derive a set of nonlinear ordinary differential equations (ODEs) that govern the motion of the L-shaped multi-beam jointed structure. The accuracy of the model is verified by the comparison of the natural frequencies solved by the frequency equation and the ANSYS. Based on the nonlinear ODEs obtained in this model, the dynamic responses are worked out to investigate the effect of the tip mass and the joint on the nonlinear dynamic characteristic of the system. The results show that the inertia of the tip mass and the nonlinear stiffness of the joints have a great influence on the nonlinear response of the system.

## 1. Introduction

A multi-beam jointed structure composed of multiple beams and flexible joints is widely used as a component on spacecrafts in the field of aerospace engineering, such as in solar arrays and large antenna support structures. The nonlinearities of the beams and the joints in the structure may have a significant effect on the dynamic characteristics of the entire system. Therefore, an in-depth study of the influence of the nonlinearities on dynamics of the system is necessary for structural design and vibration control.

As a typical multi-beam structure, the research on the dynamics of L-shaped beam structures has received extensive attention in the past few decades [1,2,3,4,5,6,7,8,9,10,11,12,13,14,15]. Because of the inertial effect of the large motion, and the geometry of motion, the L-shaped beam structure is a typical quadratic nonlinearity system. Haddow et al. [4] presented a nonlinear dynamic model of an L-shaped beam structure with two-degree-freedom and considered the effect of the axial motion of the beam. Nayfeh et al. [5,6,7] investigated the nonlinear motion of the L-shaped beam structure with lumped masses, and experimentally confirmed that the structure exhibits a chaotic response when subjected to small excitation with two-to-one internal resonance. Warminski et al. [8] formulated a detailed derivation of the nonlinear PDEs of motion for the L-shaped beam structure with different flexibilities in two orthogonal directions. Cao et al. [9] conducted theoretical and experimental studies on the periodic and chaotic motion in the L-shaped beam structure under the one-to-one internal resonance. Onozato et al. [10] investigated the chaotic responses of a post-buckled L-shaped beam with an axial constraint and examined the contribution ratio of the vibration mode to the chaotic responses by using the proper orthogonal decomposition. Taking into account the inextensionality conditions and the rotary inertia of the beam, Georgiades [11,12] derived the dynamic equations of both in-plane and out-of-plane motions for the L-shaped beam structure. Yu et al. [14,15] made a further study of the global bifurcation and the multi-pulse chaotic motion of the L-shaped beam structure by using the energy-phase method and the generalized Melnikov method.

Concerning the multi-beam structure connected with joints, there have many studies on the effect of the joint on the nonlinear dynamic behavior of the system, which mainly focuses on flexible-joint manipulators [16,17,18,19,20,21,22] and deployable structures [23,24,25,26,27,28,29]. For the flexible-joint manipulator, the joint flexibility mainly comes from the harmonic reducer. Spong et al. [16] were the first to simplify the elastic joint as a torsional spring in the dynamic modeling of the flexible-joint manipulator. By using the Euler–Lagrange principle and the assumed mode method, Subudi and Morris [17] developed a dynamic modeling approach for a manipulator with any number of flexible links and flexible joints so to derive a closed-form dynamic model suitable for vibration control. Vakil et al. [18] investigated the influence of the joint flexibility on the free vibration of the manipulator and established an upper limit for the joint stiffness so to distinguish whether the joint is flexible or rigid for the manipulator. Meng et al. [19] indicated that an appropriate stiffness of the joint can reduce the overall vibration due to the coupling between the link and joint flexibilities, which can be used as a new way to explore the vibration suppression of flexible-joint manipulators. For the manipulator joint with the planetary gear train transmission, Yang et al. [20,21] studied the effect of the time-varying stiffness, the gear tooth profile error, and the backlash clearances in the joint on the dynamics of the manipulator.

In a study of the deployable structure with joints, Moon and Li [23] experimentally studied the dynamic behavior of the pin-jointed space truss structure. The experimental results showed that very small gaps in the joints led to the chaotic motion of the structures, and that the level of chaos can be reduced by increasing the compressive load of the truss structures. Folkman et al. [24] investigated the influence of pinned joints on the damping of the truss structure with an experimental test. Bowden and Dugundji [25] proposed a geometric joint participation factor to quantify the contribution of the damping in the joints to the modal damping of the whole structure. Zhang et al. [26] presented a damping ratio formulation to investigate the effects of pressure, clearance, and dynamic parameters on the damping of joints. Guo et al. [27] systematically investigated the effects of the stiffness, the damping location, and the clearance of the joint on the dynamics of the jointed deployable structure by the finite element model and experimental method. Recently, Wei et al. [28,29] proposed a dynamic modeling approach to derive a reduced-order analytical model for a multi-beam structure with nonlinear joints. Based on this model, they studied the influence of the nonlinear stiffness and the damping of the joint on the attitude and the position of the spacecraft during maneuvering.

Moreover, the effect of the tip mass on the nonlinear dynamics of the beam structure has been investigated in Refs [30,31,32,33,34]. Esmailzadeh and Jalili [30] focused on the periodic response of the cantilever beam with the tip mass under the axial base excitation, and the results indicated that the increase of the tip mass would reduce the stable periodic region. Yabuno et al. [31] theoretically analyzed the effect of the tip mass on the bifurcation points of the frequency response curve for a parametrically excited cantilever beam. Eftekhari et al. [32] conducted a study on the primary resonance of the composite cantilever beam with the tip mass subjected to base excitation both in the flapwise and the chordwise. The results showed that the addition of the tip mass can change the dynamic stability of the system. Meesala and Hajj [34] presented a sensitivity analysis of a parametrically excited cantilever beam with the tip mass. They demonstrated that very small variations in either the stiffness or the tip mass can alter the type of bifurcation, which can potentially be applied for using parametrically excited micro-cantilever beams as sensing devices.

Although a lot of work has been done in the modeling and the analysis of the L-shaped beam structure and the multi-beam jointed structure, the research on the dynamics of the L-shaped multi-beam jointed structure with the tip mass is rarely seen. This paper is devoted to obtaining a low-dimensional nonlinear dynamics model, which is both convenient for the use of the analytical method to investigate the nonlinear dynamic behavior and suitable for real-time control. Based on the nonlinear ODEs in this model, the dynamic responses of the system can be performed to investigate the effect of the tip mass and joints on the nonlinear dynamics of the L-shaped multi-beam jointed structure, which can provide support for the analysis of the payload and the nonlinear joints of deployable structures in aerospace engineering.

## 2. Dynamical Model

### 2.1. Governing Equations of Motion

**Figure 1:**
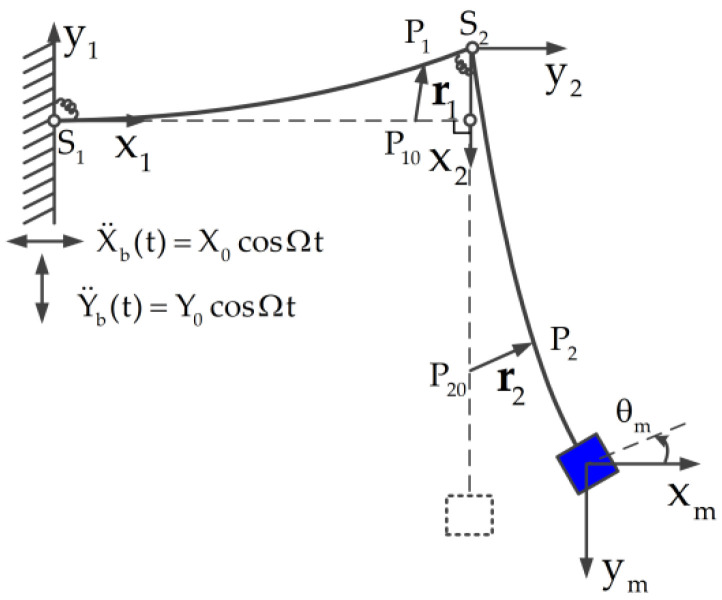
Schematic of the L-shaped multi-beam jointed structure with tip mass.

Consider the planar motion of an L-shaped multi-beam jointed structure that consists of a tip mass and two beams connected with nonlinear torsional joints, as shown in Figure 1. The horizontal beam with the length $L_{1}$ is connected to the base by the first nonlinear torsional joint $S_{1}$. Similarly, the vertical beam with the length $L_{2}$ is connected to the horizontal beam by the second nonlinear torsional joint $S_{2}$. A tip mass with mass moment of inertia J and mass m is fixed to the end of the vertical beam. Furthermore, the L-shaped multi-beam jointed structure is subjected to the horizontal acceleration ${\ddot{X}}_{b}(t)=X_{0}cos\Omega t$ and the vertical acceleration ${\ddot{Y}}_{b}(t)=Y_{0}\cos\Omega t$, where $X_{0}$ and $Y_{0}$ are the peak amplitudes of the horizontal and the vertical, respectively, and Ω is the excitation frequency. Assume that the two beams have the same dimensions as the cross-section and the same material properties. The mass per unit length, elastic modulus, and area moment of inertia of the beams are denoted by ρ,E, and I, respectively. The torsional deformation of the i-th joint $S_{i},i=1,2$ is described by $\theta_{i}$. Let $\left(x_{1},y_{1}\right)$ and $\left(x_{2},y_{2}\right)$ be the coordinates of the horizontal beam and the vertical beam, with the origin located at joint $S_{1}$ and joint $S_{2}$, respectively. Let $\left(x_{m},y_{m}\right)$ be the coordinate of the tip mass, with the origin located at the center of the tip mass. $x_{m}$ and $y_{m}$ are the translational displacements of the tip mass in the horizontal and the vertical directions, respectively. $\theta_{m}$ is used to describe the rotational displacement of the tip mass.

The deformation of arbitrary points $P_{10}$ and $P_{20}$ on the horizontal beam and the vertical beam, respectively, are depicted in Figure 1. After the deformation, the positions of $P_{10}$ and $P_{20}$ move to positions $P_{1}$ and $P_{2}$, respectively. The location vectors of positions $P_{1}$ and $P_{2}$ are given by

(1) $$r_{1}=\left[u_{1}(x,t),v_{1}(x,t)\right],\;r_{2}=\left[v_{2}(x,t),v_{1}(L_{1},t)+u_{2}(x,t)\right]$$

where $u_{1}(x,t)$ and $v_{1}(x,t)$ are the axial and transverse displacements of the horizontal beam, respectively, $v_{1}(L_{1},t)$ is the transverse displacement of the horizontal beam tip, and $u_{2}(x,t)$ and $v_{2}(x,t)$ are the axial and transverse displacements of the vertical beam, respectively. The axial displacements $u_{1}(x,t)$ and $u_{2}(x,t)$ are generally much smaller than the transverse displacements $v_{1}(x,t)$ and $v_{2}(x,t)$. Then, it is reasonable that the axial displacements $u_{1}(x,t)$ and $u_{2}(x,t)$ are neglected. Then, the location vectors of the positions $P_{1}$ and $P_{2}$ can be taken as

(2) $$r_{1}=\left[0,v_{1}(x,t)\right],\;r_{2}=\left[v_{2}(x,t),v_{1}(L_{1},t)\right]$$

Considering the effect of the axial force, the nonlinear transverse equation that governs the motion of the horizontal beam can be written as [34]

(3) $$\begin{array}{l} \rho {\ddot{v}}_{1}+c_{v}{\dot{v}}_{1}+EIv_{1}^{⁗}+\eta I{\dot{v}}_{1}^{⁗}=-EI{\left[v_{1}^{\prime }{\left(v_{1}^{\prime }v_{1}^{\prime \prime }\right)}^{\prime }\right]}^{\prime }-\frac{\rho }{2}{\left[v_{1}^{\prime }\int_{L_{1}}^{x}\int_{0}^{x}\frac{\partial^{2}}{\partial t^{2}}\left(v_{1}^{\prime }{}^{2}\right)dxdx\right]}^{\prime }\\ +\frac{m_{t}}{2}v_{1}^{\prime \prime }\int_{0}^{L_{1}}\frac{\partial^{2}}{\partial t^{2}}\left(v_{1}^{\prime }{}^{2}\right)dx+\rho {\ddot{X}}_{b}\left[v_{1}^{\prime }+\left(x-L_{1}\right)v_{1}^{\prime \prime }\right]-N_{1}v_{1}^{\prime \prime }+F_{y} \end{array}$$

where an overdot denotes partial differentiation with respect to time t, a prime denotes partial differentiation with respect to x, $c_{v}$ and η are the external and internal damping of the beam, respectively, $N_{1}=m_{t}{\ddot{X}}_{b}+\int_{0}^{L_{2}}\rho {\ddot{v}}_{2}dx+m{\ddot{x}}_{m}$ is the axial force of the horizontal beam at the joint $S_{2}$, $F_{y}=\rho {\ddot{Y}}_{b}$ is the transverse force of the horizontal beam per unit length, and $m_{t}=\rho L_{2}+m$ is the tip mass attached to the end of the horizontal beam along the x_2_ axis direction. Assume that the external and internal damping of the beam are proportional to its mass and stiffness, respectively, and they are expressed as

(4) $$c_{v}=a\rho ,\eta =bE$$

where a and b are the proportionality constant.

For the tip mass and the vertical beam, the equation that governs the motion in the x_2_ axis direction is

(5) $$\left(\rho L_{2}+m\right){\ddot{y}}_{m}+c_{r}{\dot{y}}_{m}-EIv_{1}^{\prime \prime \prime }(L_{1},t)=\left(\rho L_{2}+m\right){\ddot{Y}}_{b}$$

where $c_{r}$ is the damping coefficient of the horizontal beam and the tip mass translation in the x_2_ axis direction, and it is expressed as

(6) $$c_{r}=a(\rho L_{2}+m)$$

The nonlinear transverse equation that governs the motion of the vertical beam is

(7) $$\begin{array}{l} \rho {\ddot{v}}_{2}+c_{v}{\dot{v}}_{2}+EIv_{2}^{⁗}\;+\eta I{\dot{v}}_{2}^{⁗}\;=-EI{\left[v_{2}^{\prime }{\left(v_{2}^{\prime }v_{2}^{\prime \prime }\right)}^{\prime }\right]}^{\prime }-\frac{\rho }{2}{\left[v_{2}^{\prime }\int_{L_{2}}^{x}\int_{0}^{x}\frac{\partial^{2}}{\partial t^{2}}\left(v_{2}^{\prime }{}^{2}\right)dxdx\right]}^{\prime }\\ +\frac{m}{2}v_{2}^{\prime \prime }\int_{0}^{L_{2}}\frac{\partial^{2}}{\partial t^{2}}\left(v_{2}^{\prime }{}^{2}\right)dx+\rho \left({\ddot{Y}}_{b}+{\ddot{y}}_{m}\right)\left[v_{2}^{\prime }+\left(x-L_{2}\right)v_{2}^{\prime \prime }\right]-N_{2}v_{2}^{\prime \prime }+F_{x} \end{array}$$

where $N_{2}=m\left({\ddot{Y}}_{b}+{\ddot{y}}_{m}\right)$ is the external force of the vertical beam on the tip mass and $F_{x}=\rho {\ddot{X}}_{b}$ is the external transverse force on the vertical beam.

**Figure 2:**
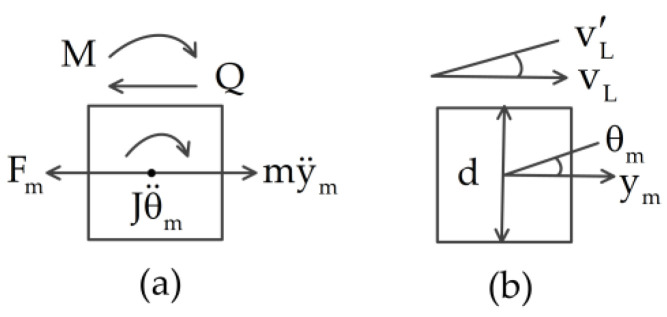
Schematic of the (**a**) natural and (**b**) geometrical matching conditions on the tip mass: (**a**) Q is the shear force of the vertical beam on the tip mass, $Q=EIv_{2}^{\prime \prime \prime }\;(L_{2},t)$, M is the bending moment of the vertical beam on the tip mass, $M=EIv_{2}^{\prime \prime }\;(L_{2},t)$, and $F_{m}$ is the external force on the tip mass; (**b**) $v_{L}=v_{2}(L_{2},t)$, $v_{2}^{\prime }=v_{2}^{\prime }(L_{2},t)$.

As for the natural matching conditions shown in Figure 2a, and according to the shear force and the bending moment of the tip mass, the equations of motion for the tip mass rotation and translation in the horizontal direction are

(8) $$J{\ddot{\theta }}_{m}+c_{J}{\dot{\theta }}_{m}+EIv_{2}^{\prime \prime }(L_{2},t)-\frac{d}{2}EIv_{2}^{\prime \prime \prime }(L_{2},t)=0$$

(9) $$m{\ddot{x}}_{m}+c_{m}{\dot{x}}_{m}-EIv_{2}^{\prime \prime \prime }(L_{2},t)=m{\ddot{X}}_{b}$$

where d is the length of the tip mass, $c_{J}$ and $c_{m}$ are the damping of the tip mass rotational and translation in the horizontal direction, respectively, and they are expressed as

(10) $$c_{J}=aJ,\;c_{m}=am$$

**Figure 3:**
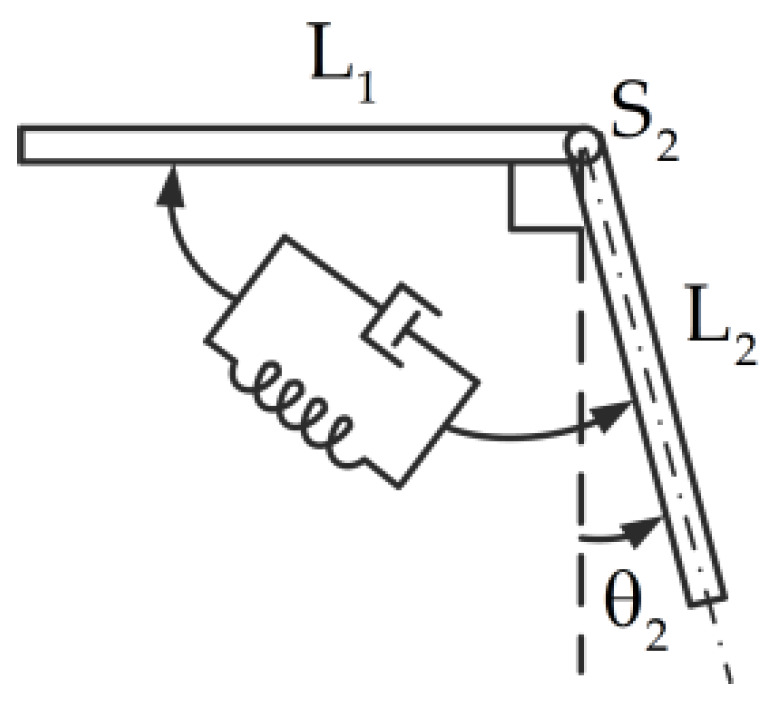
Schematic of the joint model.

The nonlinear torsional joint is described as a single-degree-of-freedom nonlinear spring massless system with a damper, as shown in Figure 3. The nonlinear transmitted torque formulation of the i-th joint can be expressed as

(11) $$M_{i}^{T}=\mu {\dot{\theta }}_{i}+k_{L}\theta_{i}+k_{N}\theta_{i}{}^{3},\;i=1,2$$

where $k_{L},k_{N},$ and μ are the linear stiffness, the cubic stiffness, and the damping of the nonlinear torsional joint, respectively.

The boundary conditions at the joint $S_{1}$ are

(12) $$v_{1}(0,t)=0,\;v_{1}^{\prime }(0,t)=\theta_{1},\;EIv_{1}^{\prime \prime }(0,t)=M_{1}^{T}$$

**Figure 4:**
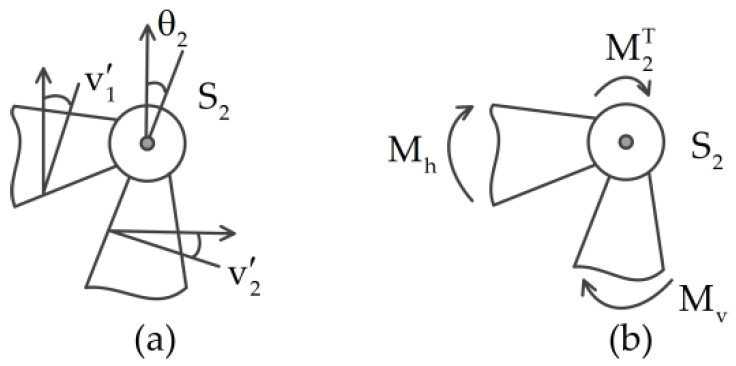
Schematic of the (**a**) geometric and (**b**) force matching conditions at the joint $S_{2}$; $v_{1}^{\prime }=v_{1}^{\prime }(L_{1},t)$, $v_{2}^{\prime }=v_{2}^{\prime }(0,t)$, $M_{h}$ and $M_{v}$ are the bending moments acting on the joint $S_{2}$, $M_{h}=EIv_{1}^{\prime \prime }(L_{1},t),\;M_{v}=EIv_{2}^{\prime \prime }(0,t)$.

As shown in Figure 4a, the geometric match conditions at the joint $S_{2}$ are

(13) $$v_{1}^{\prime }(L_{1},t)+\theta_{2}=v_{2}^{\prime }(0,t),\;v_{2}(0,t)=0$$

As shown in Figure 4b, the moment matching boundary conditions at the joint $S_{2}$ are

(14) $$EIv_{1}^{\prime \prime }(L_{1},t)=M_{2}^{T}=EIv_{2}^{\prime \prime }(0,t)$$

The geometric match conditions of the tip mass are

(15) $$v_{1}(L_{1},t)=y_{m},\;v_{2}(L_{2},t)-\frac{d}{2}\theta_{m}=x_{m},\;v_{2}^{\prime }(L_{2},t)=\theta_{m}$$

### 2.2. Determination of Natural Frequencies and Global Mode Shapes

In order to solve the eigenvalue problem of the system, it is assumed that the displacements of the L-shaped multi-beam jointed structure are separable in space and time. Let

(16) $$v_{i}(x,t)=\varphi_{i}(x)e^{j\omega t},\theta_{i}=\Theta_{i}e^{j\omega t},x_{m}=X_{m}e^{j\omega t},\;y_{m}=Y_{m}e^{j\omega t},\;\theta_{m}=\Theta_{m}e^{j\omega t},\;i=1,2$$

where ω is an unknown constant corresponding to the natural frequency of the system. Substituting the separable solutions given in Equation (16) into Equations (3), (5), (7)–(9) without damping, nonlinear terms, and external force yields

(17) $$\omega^{2}\rho \varphi_{1}(x)-EI\varphi_{1}^{⁗}(x)=0,$$

(18) $$\left(\rho L_{2}+m\right)\omega^{2}Y_{m}+EI\varphi_{1}^{\prime \prime \prime }(L_{1})=0,$$

(19) $$\omega^{2}\rho \varphi_{2}(x)-EI\varphi_{2}^{⁗}(x)=0,$$

(20) $$J\omega^{2}\Theta_{m}-EI\varphi_{2}^{\prime \prime }(L_{2})+\frac{d}{2}EI\varphi_{2}^{\prime \prime \prime }(L_{2})=0,$$

(21) $$m\omega^{2}X_{m}+EI\varphi_{2}^{\prime \prime \prime }(L_{2})=0.$$

The boundary conditions at the joints $S_{1}$ and match conditions at the joint $S_{2}$ are reduced to

(22) $$\left\{ \begin{array}{l} \varphi_{1}(0)=0,\;\varphi_{1}^{\prime }(0)=\Theta_{1},\;EI\varphi_{1}^{\prime \prime }(0)=k_{L}\Theta_{1},\\ \varphi_{2}(0)=0,\;\varphi_{1}^{\prime }(L_{1})+\Theta_{2}=\varphi_{2}^{\prime }\;(0),\\ EI\varphi_{1}^{\prime \prime }(L_{1})=k_{L}\Theta_{2},\;EI\varphi_{2}^{\prime \prime }(0)=k_{L}\Theta_{2}. \end{array}\right.$$

The geometric match conditions of the tip mass are reduced to

(23) $$\varphi_{1}(L_{1})=Y_{m},\;\varphi_{2}^{\prime }(L_{2})=\Theta_{m},\;\varphi_{2}(L_{2})-\frac{d}{2}\Theta_{m}=X_{m}.$$

The solutions of Equations (17) and (19) can be written as

(24) $$\left\{ \begin{array}{l} \varphi_{1}(x)=A_{1}\cos(\beta x)+B_{1}\sin(\beta x)+C_{1}\cosh(\beta x)+D_{1}\sinh(\beta x),\;x\in \left[0,L_{1}\right],\\ \varphi_{2}(x)=A_{2}\cos(\beta x)+B_{2}\sin(\beta x)+C_{2}\cosh(\beta x)+D_{2}\sinh(\beta x),\;x\in \left[0,L_{2}\right]. \end{array}\right.$$

where $\beta ={\left(\frac{\rho \omega^{2}}{EI}\right)}^{1/4}$. Let

(25) $$\psi ={\left[\begin{array}{ccccccccccccc} A_{1} & B_{1} & C_{1} & D_{1} & \Theta_{1} & A_{2} & B_{2} & C_{2} & D_{2} & \Theta_{2} & X_{m} & Y_{m} & \Theta_{m} \end{array}\right]}^{T}.$$

Substituting Equation (24) into the boundary and matching conditions in (22) and (23), and the dynamic equations of the tip mass in (20) and (21), yields

(26) H(ω)ψ=0,

where the matrix $H(\omega )\in R^{13\times 13}$ is given in Appendix A.

The natural frequencies of the L-shaped multi-beam jointed structure are denoted in ascending order by $\omega_{1},\omega_{2},\cdots$, which are the positive roots of the frequency equation det(H(ω))=0. Once the natural frequency $\omega_{s}$ is obtained, the eigenvector $\psi^{\left(s\right)}$ can be obtained by Equation (26). Then, the s-th global mode shapes for the L-shaped multi-beam jointed structure can be determined by Equation (24).

### 2.3. Orthogonality of the Global Mode Shapes

The global mode shapes associated with the two distinct eigenvalues $\omega_{r}$ and $\omega_{s}$ are denoted by $\phi_{\;r}(x)$ and $\phi_{\;s}(x)$, respectively, where

(27) $$\phi_{\;r}(x)={\left[\begin{array}{ccccccc} \varphi_{1r} & \Theta_{1r} & \varphi_{2r} & \Theta_{2r} & X_{mr} & Y_{mr} & \Theta_{mr} \end{array}\right]}^{T},r=1,2,\cdots .$$

By Equations (17)–(21), one has

(28) $$EI\varphi_{1r}^{⁗}(x)=\omega_{r}^{2}\rho \varphi_{1r}(x),$$

(29) $$-EI\varphi_{2r}^{⁗}(L_{1})=\omega_{r}^{2}\left(\rho L_{2}+m\right)Y_{mr},$$

(30) $$EI\varphi_{2r}^{⁗}(x)=\omega_{r}^{2}\rho \varphi_{2r}(x),$$

(31) $$EI\varphi_{2r}^{\prime \prime }(L_{2})-\frac{d}{2}EI\varphi_{2r}^{\prime \prime \prime }(L_{2})=\omega_{r}^{2}J\Theta_{mr},$$

(32) $$-EI\varphi_{2r}^{\prime \prime \prime }(L_{2})=\omega_{r}^{2}mX_{mr}.$$

Then, multiply Equations (28) and (30) by $\varphi_{1s}$ and $\varphi_{2s}$, integrate the resulting equations over the domain $0\leq x\leq L_{1}$ and $0\leq x\leq L_{2}$ for the horizontal and the vertical beams, respectively, and add the resulting equations to get

(33) $$\begin{array}{l} \int_{0}^{L_{1}}EI\varphi_{1r}^{⁗}(x)\varphi_{1s}(x)dx+\int_{0}^{L_{2}}EI\varphi_{2r}^{⁗}(x)\varphi_{2s}(x)dx=\\ \omega_{r}{}^{2}\left(\int_{0}^{L_{1}}\rho \varphi_{1r}(x)\varphi_{1s}(x)dx+\int_{0}^{L_{2}}\rho \varphi_{2r}(x)\varphi_{2s}(x)dx\right). \end{array}$$

Multiply Equations (29), (31), and (32) by $Y_{ms}$, $\Theta_{ms}$, and $X_{ms}$, respectively, and add the resulting equations to get

(34) $$\begin{array}{l} \left[EI\varphi_{2r}^{\prime \prime }(L_{2})-\frac{d}{2}EI\varphi_{2r}^{\prime \prime \prime }(L_{2})\right]\Theta_{ms}-EI\varphi_{1r}^{\prime \prime \prime }(L_{1})Y_{ms}-EI\varphi_{2r}^{\prime \prime \prime }(L_{2})X_{ms}\\ =\omega_{r}^{2}\left[\left(\rho L_{2}+m\right)Y_{mr}Y_{ms}+mX_{mr}X_{ms}+J\Theta_{mr}\Theta_{ms}\right]. \end{array}$$

Integrating Equation (33) by parts, adding Equation (34), and using the boundary and matching conditions in Equations (22) and (23) and the dynamic equations in (18), (20), and (21) yields

(35) $$\begin{array}{l} \int_{0}^{L_{1}}EI\varphi_{1r}^{⁗}(x)\varphi_{1s}(x)dx+\int_{0}^{L_{2}}EI\varphi_{2r}^{⁗}(x)\varphi_{2s}(x)dx=\\ \int_{0}^{L_{1}}EI\varphi_{1r}^{\prime \prime }(x)\varphi_{1S}^{\prime \prime }(x)dx+\int_{0}^{L_{2}}EI\varphi_{2r}^{\prime \prime }(x)\varphi_{2s}^{\prime \prime }(x)dx+k_{L}\left(\Theta_{1r}\Theta_{1s}+\Theta_{2r}\Theta_{2s}\right)\\ -\omega_{r}^{2}\left[\left(\rho L_{2}+m\right)Y_{mr}Y_{ms}+mX_{mr}X_{ms}+J\Theta_{mr}\Theta_{ms}\right]. \end{array}$$

Substituting Equation (35) into the left-hand side of Equation (33) yields

(36) $$\begin{array}{l} \int_{0}^{L_{1}}EI\varphi_{1r}^{\prime \prime }(x)\varphi_{1s}^{\prime \prime }(x)dx+\int_{0}^{L_{2}}EI\varphi_{2r}^{\prime \prime }(x)\varphi_{2s}^{\prime \prime }(x)dx+k_{L}\left(\Theta_{1r}\Theta_{1s}+\Theta_{2r}\Theta_{2s}\right)=\\ \omega_{r}{}^{2}\left[\begin{array}{l} \int_{0}^{L_{1}}\rho \varphi_{1r}(x)\varphi_{1s}(x)dx+\int_{0}^{L_{2}}\rho \varphi_{2r}(x)\varphi_{2s}(x)dx\\ +\left(\rho L_{2}+m\right)Y_{mr}Y_{ms}+mX_{mr}X_{ms}+J\Theta_{mr}\Theta_{ms} \end{array}\right]. \end{array}$$

Exchanging the superscripts s and r in Equation (36) yields

(37) $$\begin{array}{l} \int_{0}^{L_{1}}EI\varphi_{1r}^{\prime \prime }(x)\varphi_{1s}^{\prime \prime }(x)dx+\int_{0}^{L_{2}}EI\varphi_{2r}^{\prime \prime }(x)\varphi_{2s}^{\prime \prime }(x)dx+k_{L}\left(\Theta_{1r}\Theta_{1s}+\Theta_{2r}\Theta_{2s}\right)=\\ \omega_{s}{}^{2}\left[\begin{array}{l} \int_{0}^{L_{1}}\rho \varphi_{1r}(x)\varphi_{1s}(x)dx+\int_{0}^{L_{2}}\rho \varphi_{2r}(x)\varphi_{2s}(x)dx\\ +\left(\rho L_{2}+m\right)Y_{mr}Y_{ms}+mX_{mr}X_{ms}+J\Theta_{mr}\Theta_{ms} \end{array}\right]. \end{array}$$

Subtracting Equation (37) from Equation (36) yields

(38) $$\begin{array}{l} (\omega_{r}{}^{2}-\omega_{s}{}^{2})\left[\int_{0}^{L_{1}}\rho \varphi_{1r}(x)\varphi_{1s}(x)dx+\int_{0}^{L_{2}}\rho \varphi_{2r}(x)\varphi_{2s}(x)dx\right]+\\ (\omega_{r}{}^{2}-\omega_{s}{}^{2})\left[\left(\rho L_{2}+m\right)Y_{mr}Y_{ms}+mX_{mr}X_{ms}+J\Theta_{mr}\Theta_{ms}\right]=0. \end{array}$$

From Equation (38), the first orthogonality relation can be obtained

(39) $$\begin{array}{l} \int_{0}^{L_{1}}\rho \varphi_{1r}(x)\varphi_{1s}(x)dx+\int_{0}^{L_{2}}\rho \varphi_{2r}(x)\varphi_{2s}(x)dx+\\ \left(\rho L_{2}+m\right)Y_{mr}Y_{ms}+mX_{mr}X_{ms}+J\Theta_{mr}\Theta_{ms}=M_{s}\delta_{rs}, \end{array}$$

where $M_{s}$ is a positive constant and $\delta_{rs}$ is the Kronecker delta. Using Equations (36) and (39), the second orthogonality relation can be obtained

(40) $$\int_{0}^{L_{1}}EI\varphi_{1r}^{\prime \prime }(x)\varphi_{1s}^{\prime \prime }(x)dx+\int_{0}^{L_{2}}EI\varphi_{2r}^{\prime \prime }(x)\varphi_{2s}^{\prime \prime }(x)dx+k_{L}\left(\Theta_{1r}\Theta_{1s}+\Theta_{2r}\Theta_{2s}\right)=K_{s}\delta_{rs}.$$

where $K_{s}$ is a positive constant.

### 2.4. Dynamic Model with Multi-DOF

Applying the Galerkin procedure to Equations (3), (5), and (7)–(9), the nonlinear ODEs are derived for the system. By using the first n global mode shapes, the displacements of the L-shaped multi-beam jointed structure can be written as

(41) $$\begin{array}{l} v_{1}(x,t)=\sum_{j=1}^{n}\varphi_{1j}(x)q_{j}(t),\;v_{2}(x,t)=\sum_{j=1}^{n}\varphi_{2j}(x)q_{j}(t),\;\theta_{1}=\sum_{j=1}^{n}\Theta_{1j}q_{j}(t),\\ \theta_{2}=\sum_{j=1}^{n}\Theta_{2j}q_{j}(t),\;x_{m}=\sum_{j=1}^{n}X_{mj}q_{j}(t),\;y_{m}=\sum_{j=1}^{n}Y_{mj}q_{j}(t),\;\theta_{m}=\sum_{j=1}^{n}\Theta_{mj}q_{j}(t). \end{array}$$

where $q_{j}(t)$ is the j-th modal coordinate of the system.

Substituting Equation (41) into Equations (3), (5), (7)–(9), and (11) yields

(42) $$\begin{array}{l} \sum_{j=1}^{n}\rho \varphi_{1j}{\ddot{q}}_{j}+\sum_{j=1}^{n}c_{v}\varphi_{1j}{\dot{q}}_{j}+\sum_{j=1}^{n}EI\varphi_{1j}^{⁗}q_{j}+\sum_{j=1}^{n}\eta I\varphi_{1j}^{⁗}{\dot{q}}_{j}=\sum_{j=1}^{n}\rho {\ddot{X}}_{b}\left[{\varphi^{\prime }}_{1j}+\left(x-L_{1}\right)\varphi_{1j}^{\prime \prime }\right]q_{j}\\ -\sum_{j=1}^{n}\sum_{k=1}^{n}\sum_{r=1}^{n}EI{\left[\varphi_{1j}^{\prime }{\left(\varphi_{1k}^{\prime }\varphi_{1r}^{\prime \prime }\right)}^{¢}\right]}^{¢}q_{j}q_{k}q_{r}\\ -{\sum_{j=1}^{n}\sum_{k=1}^{n}\sum_{r=1}^{n}\rho \left\{\varphi_{1j}^{\prime }q_{j}\int_{L_{1}}^{x}\int_{0}^{x}\left[\varphi_{1k}^{\prime }\varphi_{1r}^{\prime }\left({\dot{q}}_{k}{\dot{q}}_{r}+{\dot{q}}_{k}{\ddot{q}}_{r}\right)\right]dxdx\right\}}^{\prime }\\ +\sum_{j=1}^{n}\sum_{k=1}^{n}\sum_{r=1}^{n}\left(\rho L_{2}+m\right)\varphi_{1j}^{\prime \prime }q_{j}\int_{0}^{L_{1}}\left[\varphi_{1k}^{\prime }\varphi_{1r}^{\prime }\left({\dot{q}}_{k}{\dot{q}}_{r}+{\dot{q}}_{k}{\ddot{q}}_{r}\right)\right]dx\\ -\sum_{j=1}^{n}\left(\rho L_{2}+m\right){\ddot{X}}_{b}\varphi_{1j}^{\prime \prime }q_{j}+\sum_{j=1}^{n}\sum_{k=1}^{n}\left(\int_{0}^{L_{2}}\rho \varphi_{2k}dx+mX_{mk}\right){\ddot{q}}_{k}\varphi_{1j}^{\prime \prime }q_{j}+\rho {\ddot{Y}}_{b}, \end{array}$$

(43) $$\sum_{j=1}^{n}\left(\rho L_{2}+m\right)Y_{mj}{\ddot{q}}_{j}+\sum_{j=1}^{n}c_{r}Y_{mj}{\dot{q}}_{j}-\sum_{j=1}^{n}EI\varphi_{1j}^{\prime \prime \prime }q_{j}=\left(\rho L_{2}+m\right){\ddot{Y}}_{b},$$

(44) $$\begin{array}{l} \sum_{j=1}^{n}\rho \varphi_{2j}{\ddot{q}}_{j}+\sum_{j=1}^{n}c_{v}\varphi_{2j}{\dot{q}}_{j}+\sum_{j=1}^{n}EI\varphi_{2j}^{⁗}q_{j}+\sum_{j=1}^{n}\eta I\varphi_{2j}^{⁗}{\dot{q}}_{j}=\\ -\sum_{j=1}^{n}\sum_{k=1}^{n}\sum_{r=1}^{n}EI{\left[\varphi_{2j}^{\prime }{\left(\varphi_{2k}^{\prime }\varphi_{2r}^{\prime \prime }\right)}^{¢}\right]}^{\prime }q_{j}q_{k}q_{r}+\sum_{j=1}^{n}\sum_{k=1}^{n}\rho Y_{mk}\left[\varphi_{2j}^{\prime }+\left(x-L_{2}\right)\varphi_{2j}^{\prime \prime }\right]q_{j}{\ddot{q}}_{k}\\ -\sum_{j=1}^{n}\sum_{k=1}^{n}\sum_{r=1}^{n}\rho {\left\{\varphi_{2j}^{\prime }q_{j}\int_{L_{2}}^{x}\int_{0}^{x}\left[\varphi_{2k}^{\prime }\varphi_{2r}^{\prime }\left({\dot{q}}_{k}{\dot{q}}_{r}+{\dot{q}}_{k}{\ddot{q}}_{r}\right)\right]dxdx\right\}}^{\prime }\\ +\sum_{j=1}^{n}\sum_{k=1}^{n}\sum_{r=1}^{n}m\varphi_{2j}^{\prime \prime }q_{j}\int_{0}^{L_{2}}\left[\varphi_{2k}^{\prime }\varphi_{2r}^{\prime }\left({\dot{q}}_{k}{\dot{q}}_{r}+{\dot{q}}_{k}{\ddot{q}}_{r}\right)\right]dx-\sum_{j=1}^{n}\sum_{k=1}^{n}mY_{mk}\varphi_{2j}^{\prime \prime }q_{j}{\ddot{q}}_{k}\\ +\sum_{j=1}^{n}\rho {\ddot{Y}}_{b}\left[\varphi_{2j}^{\prime }+\left(x-L_{2}\right)\varphi_{2j}^{\prime \prime }\right]q_{j}-\sum_{j=1}^{n}m{\ddot{Y}}_{b}\varphi_{2j}^{\prime \prime }q_{j}+\rho {\ddot{X}}_{b}, \end{array}$$

(45) $$\sum_{j=1}^{n}J\Theta_{mj}{\ddot{q}}_{j}+\sum_{j=1}^{n}c_{J}\Theta_{mj}{\dot{q}}_{j}+\sum_{j=1}^{n}EI\varphi_{2j}^{\prime \prime }(L_{2})q_{j}-\frac{d}{2}\sum_{j=1}^{n}EI\varphi_{2j}^{\prime \prime \prime }(L_{2})q_{j}=0,$$

(46) $$\sum_{j=1}^{n}mX_{mj}{\ddot{q}}_{j}+\sum_{j=1}^{n}c_{m}X_{mj}{\dot{q}}_{j}-\sum_{j=1}^{n}EI\varphi_{2j}^{\prime \prime \prime }q_{j}=m{\ddot{X}}_{b},$$

(47) $$M_{i}^{T}=\sum_{j=1}^{n}\mu \Theta_{ij}{\dot{q}}_{j}+\sum_{j=1}^{n}k_{L}\Theta_{ij}q_{j}+\sum_{j=1}^{n}\sum_{k=1}^{n}\sum_{r=1}^{n}k_{N}\Theta_{ij}\Theta_{ik}\Theta_{ir}q_{j}q_{k}q_{r},\;i=1,2.$$

Multiplying Equations (42) and (44) by $\varphi_{1s}$ and $\varphi_{2s}$, integrating the resulting equations over the domain $0\leq x\leq L_{1}$ and $0\leq x\leq L_{2}$ for the horizontal beam and the vertical beam, multiplying Equations (43), (45), and (46) by $Y_{ms}$, $\Theta_{ms}$, and $X_{ms}$, respectively, and adding all of the resulting equations and using the matching and boundary conditions in Equations (12)–(15) and the orthogonality relations in Equations (39) and (40), we have

(48) $$\begin{array}{l} {\ddot{q}}_{s}+\omega_{s}^{2}q_{s}+2\alpha_{s}\omega_{s}{\dot{q}}_{s}+\sum_{j=1}^{n}\gamma_{j}^{s}{\dot{q}}_{j}+\sum_{j=1}^{n}a_{j}^{s}q_{j}+\sum_{j=1}^{n}\sum_{k=1}^{n}b_{jk}^{s}q_{j}{\ddot{q}}_{k}+\sum_{j=1}^{n}\sum_{k=1}^{n}\sum_{r=1}^{n}c_{jkr}^{s}q_{j}q_{k}q_{r}\\ +\sum_{j=1}^{n}\sum_{k=1}^{n}\sum_{r=1}^{n}d_{jkr}^{s}\left(q_{j}q_{k}{\ddot{q}}_{r}+q_{j}{\dot{q}}_{k}{\dot{q}}_{r}\right)=f_{s}(t),\;s=1,2,\cdots ,n. \end{array}$$

where $\alpha_{s}$ and $\gamma_{j}^{s}$ are the damping of the beams and joints, respectively. The terms $a_{j}^{s}$ are resulted from the axial force of the beams caused by the foundation motion, the terms $b_{jk}^{s}$ are resulted from the axial forces of the beams caused by the elastic motion of the beams, the terms $c_{jkr}^{s}$ represent the geometric nonlinearities of the beams and the nonlinear stiffness of the joints, and the terms $d_{jkr}^{s}$ represent the inertial nonlinearities of the beams and the tip mass. The relevant terms in Equation (48) are given in Appendix B.

## 3. Results and Discussion

In this section, a comparison of the natural frequencies obtained from the frequency equation and the finite element software ANSYS (Version 16.0, ANSYS Inc, Canonsburg, PA, USA) is presented so to verify the accuracy of the model, and the first four global mode shapes of the L-shaped multi-beam structure are also given. Then, the dynamic responses of the system with various parameters are worked out to investigate the effect of the tip mass and the joints on the nonlinear vibration of the L-shaped multi-beam structure under base excitation. The physical parameters of the L-shaped multi-beam structure are listed in Table 1.

### 3.1. Mode Analysis

**Figure 5:**
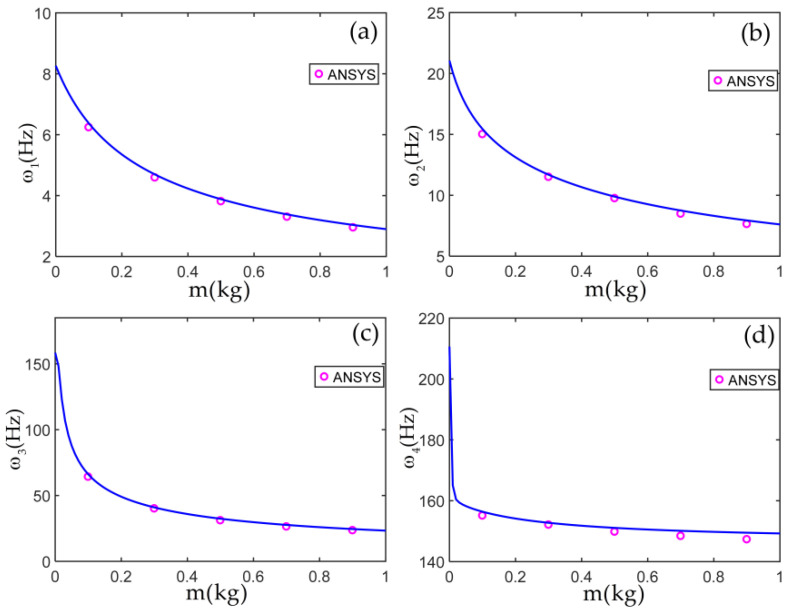
The first four natural frequencies versus m: (**a**–**d**) 1st to 4th natural frequencies.

To verify the accuracy of the dynamic model obtained in this paper, the first four natural frequencies of the L-shaped multi-beam structure with various tip mass parameters are solved by the frequency equation det(H(ω))=0 and compared with those obtained from the finite element software ANSYS, as shown in Figure 5. For the finite element model, the beam, the flexible joint, and the tip mass are modeled by the BEAM element, the COMBIN 14 element, and the MASS element, respectively. Each beam is divided into 300 elements. The joint linear torsional stiffness is selected as $k_{L}=EI/L$, and the moment of inertia of the tip mass increases in proportion to its mass. The pink symbol ○ represents the results from the finite element software ANSYS.

**Figure 6:**
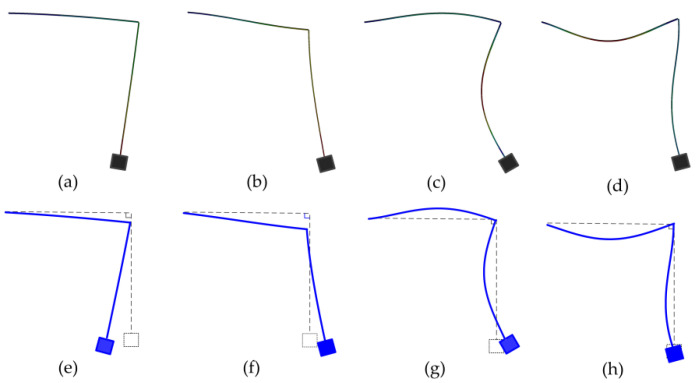
The 1st to 4th global mode shapes of the structure from ANSYS (**a**–**d**) and the presented model (**e**–**h**).

As shown in Figure 5, the results, solved by the frequency equation from m=0 kg to m=1 kg are in good agreement with those by the finite element software ANSYS. This demonstrates the correctness of the dynamic model in this paper. The first four natural frequencies and the global mode shapes of the L-shaped multi-beam jointed structure with m=0.5 kg are illustrated in Figure 6.

### 3.2. Nonlinear Responses

**Figure 7:**
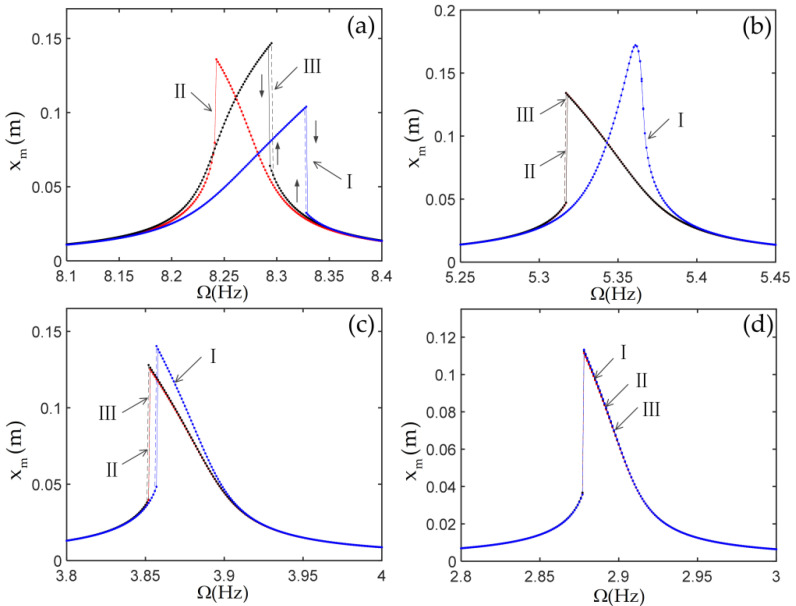
The frequency response curves of horizontal displacement of the tip mass: (**a**) m=0,J=0; (**b**) m=0.2,J=0.004; (**c**) m=0.5,J=0.01; (**d**) m=1,J=0.02.

**Figure 8:**
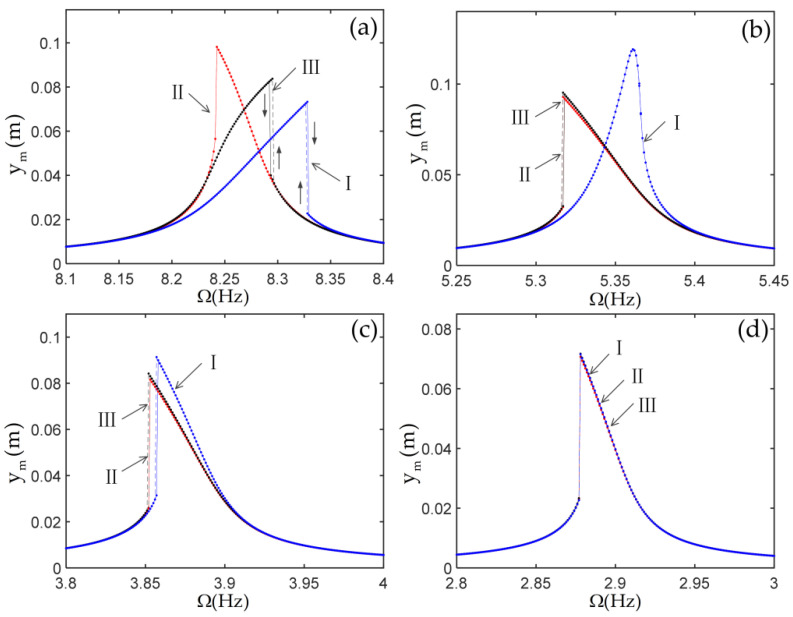
The frequency response curves of vertical displacement of the tip mass: (**a**) m=0,J=0; (**b**) m=0.2,J=0.004; (**c**) m=0.5,J=0.01; (**d**) m=1,J=0.02.

As the tip mass is fixed to the end of the vertical beam, it will cause a change of axial force, geometric nonlinearity, and inertial nonlinearity, and then it will affect the nonlinear dynamic response of the system. To investigate the effect of the tip mass on the nonlinear response of the system, the frequency response curves of the tip mass with various parameters are calculated by the MATLAB (Version 2017a, MathWorks, Natick, MA, USA) ode function derived from the Runge–Kutta method, as shown in Figure 7 and Figure 8. In these figures, the roman numerals I represent the results of considering only the geometric nonlinearity of the beams, which indicates that the terms $a_{j}^{s},b_{jk}^{s}$ and $d_{jkr}^{s}$ are missed in the case I. The roman numerals II represent the results of considering only the geometric nonlinearity of the beams, the inertial nonlinearity, and the axial force of the beams caused by the foundation motion, which indicates that the terms $b_{jk}^{s}$ are missed in this case II. The roman numerals III represent the results from the Equation (48), which includes all nonlinear terms. In Figure 7 and Figure 8, the damping and the nonlinear stiffness of the joints are selected as $\mu =0,k_{N}=0$.

From Figure 6, it is observed that the second natural frequency is much smaller than the third natural frequency. Then, one can select only the first two modes in calculating the dynamic responses of the system when Ω varies near the first natural frequency of the L-shaped multi-beam jointed structure. The results with increasing and decreasing Ω are obtained as follows: the initial conditions for the first point are assumed to be zero, and some steady-state response data at a current Ω are used as the initial conditions for the next Ω.

**Figure 9:**
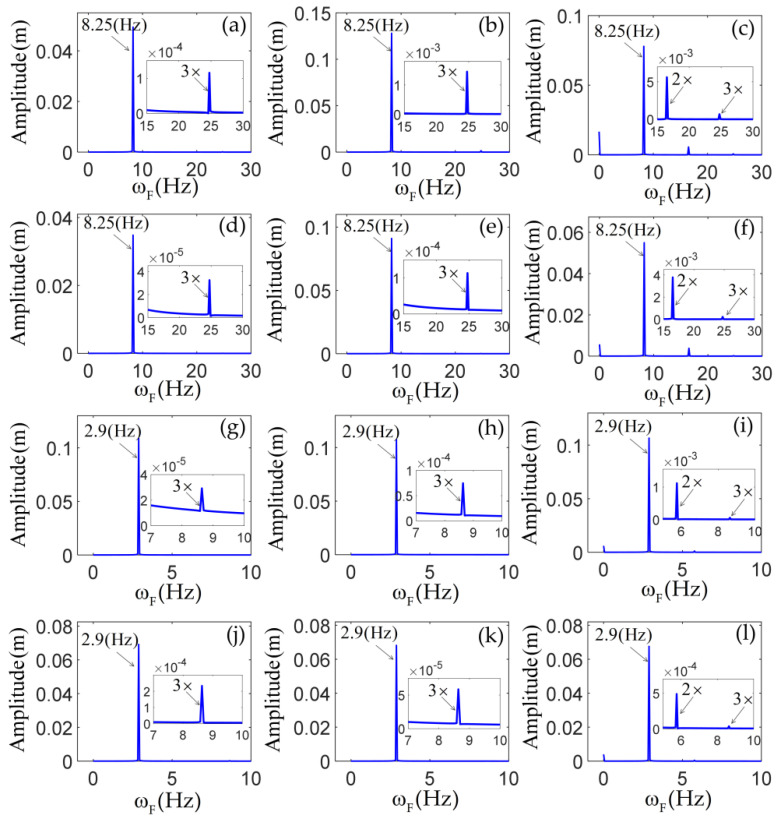
The spectrum plots of the steady-state responses of the tip mass: the horizontal displacement (**a**–**c**) and the vertical displacement (**d**–**f**) with m=0,J=0 at Ω=8.25  Hz; The horizontal displacement (**g**–**i**) and the vertical displacement (**j**–**l**) with m=1,J=0.02 at Ω=8.25Hz; (**a**,**d**,**g,j**) the results from the case I; (**b,e**,**h**,**k**) the results from the case II; (**c**,**f**,**I**,**l**) the results from the case III.

The spectrum plots of the steady-state response of the tip mass in the cases of m=0,J=0 and m=1,J=0.02 are shown in Figure 9. From this figure, it can be observed that the terms $d_{jkr}^{s}$ resulted from the inertial nonlinearity, the terms $c_{jkr}^{s}$ resulted from the geometric nonlinearity of the beams, which led to the generation of the third harmonics, and the terms $b_{jk}^{s}$ resulted from the axial force of the beams caused by the elastic motion, which led to the generation of the second harmonics. It is important to note that the terms $b_{jk}^{s}$ led to a zero frequency in the spectrum.

**Figure 10:**
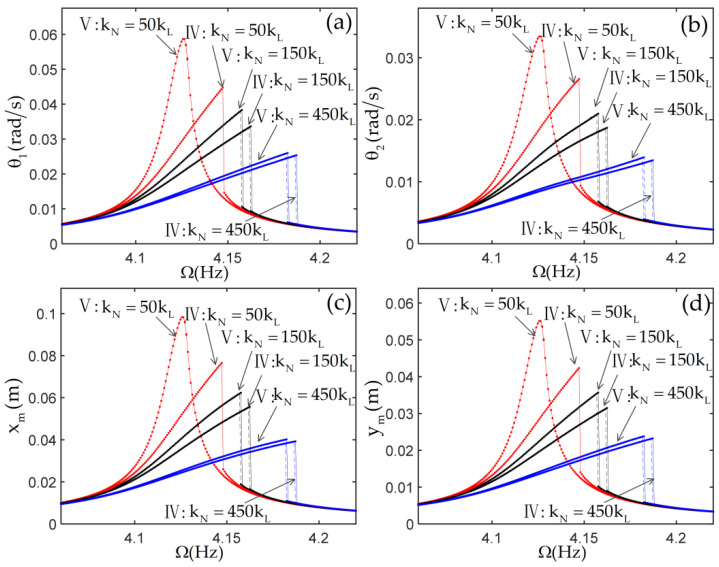
The frequency response curves of the structure with $k_{L}=5EI/L$. (**a**) the displacement of first joint; (**b**) the displacement of second joint; (**c**) the horizontal displacement of tip mass; (**d**) the vertical displacement of tip mass.

**Figure 11:**
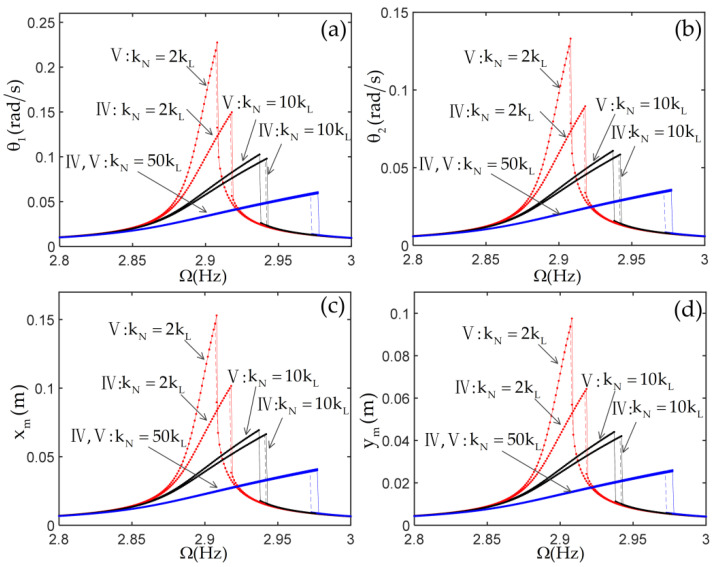
The frequency response curves of the structure with $k_{L}=EI/L$. (**a**) the displacement of first joint; (**b**) the displacement of second joint; (**c**) the horizontal displacement of tip mass; (**d**) the vertical displacement of tip mass.

To investigate the effect of the nonlinear stiffness of the joint and the nonlinearities from the beams on the nonlinear responses of the system, the frequency response curves of the system with various linear stiffnesses $k_{L}$ and nonlinear stiffnesses $k_{N}$ are given in Figure 10 and Figure 11. The tip mass is selected as m=1,J=0.02. The joint linear stiffness is selected as $k_{L}=5EI/L$ and $k_{L}=EI/L$ in Figure 10 and Figure 11, respectively. In these figures, the roman numerals IV represent the results of considering only the nonlinearities of the joints. The roman numerals V represent the results of considering the nonlinearities of the joints and the nonlinearities from the beams, Equation (48), and including all nonlinear terms. 

To investigate the effect of the nonlinear stiffness of the joint and the nonlinearities from the beams on the nonlinear responses of the system, the frequency response curves of the system with various linear stiffnesses $k_{L}$ and nonlinear stiffnesses $k_{N}$ are given in Figure 10 and Figure 11. The tip mass is selected as m=1,J=0.02. The joint linear stiffness is selected as $k_{L}=5EI/L$ and $k_{L}=EI/L$ in Figure 10 and Figure 11, respectively. In these figures, the roman numerals IV represent the results of considering only the nonlinearities of the joints. The roman numerals V represent the results of considering the nonlinearities of the joints and the nonlinearities from the beams, Equation (48), and including all nonlinear terms.

In the case of m=1,J=0.02, the frequency response of the system shows soft characteristics when the joint nonlinear stiffness is selected as $k_{N}=0$. In Figure 10 and Figure 11, it is clearly seen that the frequency response of the system shows hard characteristics due to the addition of the positive cubic nonlinear stiffness of the joint. As the positive cubic nonlinear stiffness of the joint increases, the response amplitude of the system decreases gradually, and the jump occurs at a higher frequency. By comparing the results from two cases, IV and V, it can be observed that the differences obtained from the two cases become smaller and smaller. This shows that the cubic nonlinear stiffness of the joints gradually becomes the main source of the system’s nonlinearity with the increase of the positive cubic nonlinear stiffness of the joints. By comparing Figure 10 with Figure 11, when the linear stiffness of the joint is relatively small, the cubic nonlinear stiffness of the joint has a great influence on the frequency response of the system because of its large joint torsion deformation, and the influence of other nonlinearities is small or even negligible. For instance, the very small differences in the frequency responses obtained from the two cases show that the nonlinear dynamic characteristics of the system are dominated by the nonlinear stiffness of the joint in Figure 11d.

**Figure 12:**
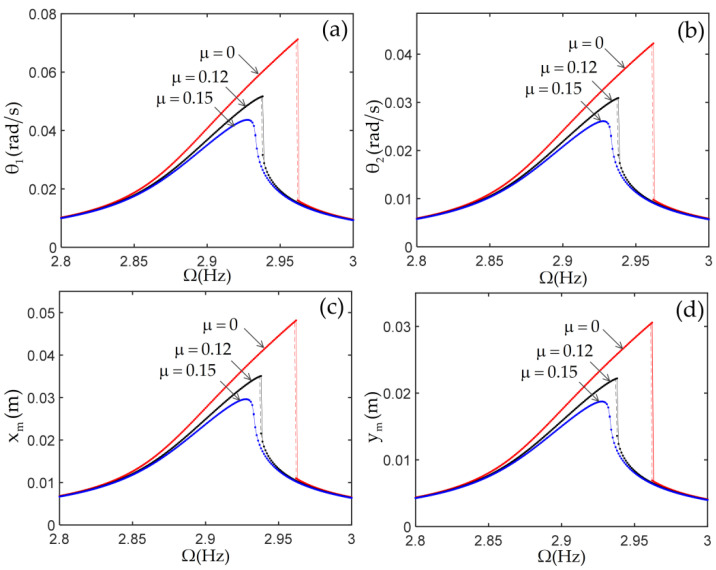
The frequency response curves of the structure with various joint damping μ. (**a**) the displacement of first joint; (**b**) the displacement of second joint; (**c**) the horizontal displacement of tip mass; (**d**) the vertical displacement of tip mass.

The frequency response curves of the structure with various joint dampings μ are shown in Figure 12. From this figure, it is clearly seen that, in the case without joint damping, the jump phenomenon is very obvious. With the increase of joint damping, the vibration amplitude of the tip mass decreases, and the jump phenomenon gradually diminishes until it disappears.

## 4. Conclusions

In this paper, by taking into account the axial force and the geometric nonlinearities of the beams, the nonlinear stiffness of the joints, and the inertial nonlinearities, a nonlinear dynamic model of an L-shaped multi-beam jointed structure has been developed by using the dynamic modeling approach proposed in Ref [28]. A comparison of the natural frequencies solved by the frequency equation and the ANSYS has been given to verify the accuracy of the model in this paper. Based on the nonlinear ODEs obtained in this model, the dynamic responses of the L-shaped multi-beam jointed structure have been presented to investigate the effect of the tip mass and the joints on the nonlinear dynamics of the system. Two conclusions are summarized as follows:

(1) In the case without the nonlinear stiffness of the joints, as the inertia of the tip mass increases, the geometric nonlinearity of the beam causes the frequency responses to change from showing the hard characteristics to showing the soft characteristics. The large inertial of the tip mass results in the frequency responses of the system without the joint nonlinearities to be dominated by the geometric nonlinearities of the beams. In addition, the axial force of the beams caused by the elastic motion leads to a zero frequency in the spectrum.

(2) With the addition of the positive cubic nonlinear stiffness of the joint, the response amplitude of the system decreases, and the jump occurs at a higher frequency. When the torsional deformation of the joint is relatively large, the nonlinear stiffness of the joints has a great influence on the frequency response of the system, and the influence of other nonlinearities is small or even negligible.

In summary, the nonlinear dynamic model derived in this paper can guarantee accuracy while at the same time greatly reduce its degree of freedom. This makes the model not only convenient for the analytical method to better understand the nonlinear dynamic behavior of the system, but also to reduce the calculation of the dynamic analysis in structural design. More importantly, this model is very suitable for real-time control due to its fewer degrees of freedom. All of these are of great significance to nonlinear dynamic analysis and the active vibration control of the multi-beam jointed structure.

## Figures and Tables

**Table 1:** Values of the parameters of the L-shaped multi-beam jointed structure.

Parameter	Value
$\mathrm{Beam}\;\mathrm{density}\;\rho \;({\mathrm{kg}/m}^{3})$	7800
Beam elastic modulus E (GPa)	200
Beam width (m)	0.02
Beam thickness (m)	0.005
$\mathrm{Beam}\;\mathrm{length}\;L_{1}=L_{2}=L$ (m)	0.3
$\mathrm{Mass}\;\mathrm{moment}\;\mathrm{inertia}\;\mathrm{of}\;\mathrm{tip}\;\mathrm{mas}\;J\;(\mathrm{kg}\cdot m^{2})$	0.01
$\mathrm{Joint}\;\mathrm{linear}\;\mathrm{stiffness}\;k_{L}(N\cdot m/\mathrm{rad})$	138.5

## Data Availability

Not applicable.

## Appendix A

Entries of the matrix H in Equation (26)

$$\begin{array}{l} H_{1,1}=\cos(0),H_{1,2}=\sin(0),H_{1,3}=\cosh(0),H_{1,4}=\sinh(0),\\ H_{2,1}=-\beta \sin(0),H_{2,2}=\beta \cos(0),H_{2,3}=\beta \sinh(0),H_{2,4}=\beta \cosh(0),\\ H_{3,1}=-EI\beta^{2}\cos(0),H_{3,2}=-EI\beta^{2}\sin(0),H_{3,3}=EI\beta^{2}\cosh(0),H_{3,4}=EI\beta^{2}\sinh(0),\\ H_{3,5}=-k_{1},H_{4,6}=\cos(0),H_{4,7}=\sin(0),H_{4,8}=\cosh(0),H_{4,9}=\sinh(0),\\ H_{5,1}=-\beta \sin(\beta L_{1}),H_{5,2}=\beta \cos(\beta L_{1}),H_{5,3}=\beta \sinh(\beta L_{1}),H_{5,4}=\beta \cosh(\beta L_{1}),\\ H_{5,6}=\beta \sin(0),H_{5,7}=-\beta \cos(0),H_{5,8}=-\beta \sinh(0),H_{5,9}=-\beta \cosh(0),H_{5,10}=1,\\ H_{6,1}=-EI\beta^{2}\cos(\beta L_{1}),H_{6,2}=-EI\beta^{2}\sin(\beta L_{1}),H_{6,3}=EI\beta^{2}\cosh(\beta L_{1}),\\ H_{6,4}=EI\beta^{2}\sinh(\beta L_{1}),H_{6,5}=-k_{1},H_{7,5}=-k_{1},H_{7,6}=-EI\beta^{2}\cos(0),\\ H_{7,7}=-EI\beta^{2}\sin(0),H_{7,8}=EI\beta^{2}\cosh(0),H_{7,9}=EI\beta^{2}\sinh(0),\\ H_{8,1}=EI\beta^{3}\sin(\beta L_{1}),H_{8,2}=-EI\beta^{3}\cos(\beta L_{1}),H_{8,3}=EI\beta^{3}\sinh(\beta L_{1}),\\ H_{8,4}=EI\beta^{3}\cosh(\beta L_{1}),H_{8,12}=(\rho L_{2}+m)\omega^{2},H_{9,1}=\cos(\beta L_{1}),\\ H_{9,2}=\sin(\beta L_{1}),H_{9,3}=\cosh(\beta L_{1}),H_{9,4}=\sinh(\beta L_{1}),H_{9,12}=-1,H_{10,6}=-\beta \sin(\beta L_{2}),\\ H_{10,7}=\beta \cos(\beta L_{2}),H_{10,8}=\beta \sinh(\beta L_{2}),H_{10,9}=\beta \cosh(\beta L_{2}),H_{10,12}=-1,\\ H_{11,6}=\cos(\beta L_{2}),H_{11,7}=\sin(\beta L_{2}),H_{11,8}=\cosh(\beta L_{2}),H_{11,9}=\sinh(\beta L_{2}),H_{11,10}=-1,\\ H_{11,13}=-d/2,H_{12,6}=EI\beta^{3}\sin(\beta L_{2}),H_{12,7}=-EI\beta^{3}\cos(\beta L_{2}),\\ H_{12,8}=EI\beta^{3}\sinh(\beta L_{2}),H_{12,9}=EI\beta^{3}\cosh(\beta L_{2}),H_{12,10}=m\omega^{2},H_{13,13}=J\omega^{2},\\ H_{13,6}=EI\beta^{2}\cos(\beta L_{2})+dEI\beta^{3}\sin(\beta L_{2})/2,H_{13,7}=EI\beta^{2}\sin(\beta L_{2})-dEI\beta^{3}\cos(\beta L_{2})/2,\\ H_{13,8}=-EI\beta^{2}\cosh(\beta L_{2})+dEI\beta^{3}\sinh(\beta L_{2})/2,H_{13,9}=-EI\beta^{2}\sinh(\beta L_{2})+dEI\beta^{3}\cosh(\beta L_{2})/2, \end{array}$$

where $H_{i,j}$ represents the i-th row and j-th columns of the matrix H, and all the other elements of the matrix H are zero.

## Appendix B

The relevant terms in Equation (48)

$$\begin{array}{l} M_{s}=\int_{0}^{L_{1}}\rho \varphi_{1s}{}^{2}dx+\int_{0}^{L_{2}}\rho \varphi_{2s}{}^{2}dx+\left(\rho L_{2}+m\right)Y_{ms}{}^{2}+mX_{ms}{}^{2}+J\Theta_{ms}{}^{2},\\ K_{s}=\int_{0}^{L_{1}}EI\varphi_{1s}^{\prime \prime }{}^{2}dx+\int_{0}^{L_{2}}EI\varphi_{2s}^{\prime \prime }{}^{2}dx+k_{L}\left(\Theta_{1s}{}^{2}+\Theta_{2s}{}^{2}\right),\\ \omega_{s}^{2}=\frac{K_{s}}{M_{s}},\;\alpha_{s}=\frac{1}{2}\left(\frac{a}{\omega_{s}}+b\omega_{s}\right),\;\gamma_{j}^{s}=\frac{\mu }{M_{s}}\left(\Theta_{1j}\Theta_{1s}+\Theta_{2j}\Theta_{2s}\right),\;\\ a_{j}^{s}=\frac{1}{M_{s}}\left\{\begin{array}{l} -{\ddot{X}}_{b}\int_{0}^{L_{1}}\varphi_{1s}\left[\rho \left(\varphi_{1j}^{\prime }+\left(x-L_{1}\right)\varphi_{1j}^{\prime \prime }\right)-\left(\rho L_{2}+m\right)\varphi_{1j}^{\prime \prime }\right]dx\\ -{\ddot{Y}}_{b}\int_{0}^{L_{2}}\varphi_{2s}\left[\rho \left(\varphi_{2j}^{\prime }+\left(x-L_{2}\right)\varphi_{2j}^{\prime \prime }\right)-m\varphi_{2j}^{\prime \prime }\right]dx \end{array}\right\},\\ b_{jk}^{s}=\frac{1}{M_{s}}\left\{\begin{array}{l} \int_{0}^{L_{1}}\varphi_{1s}\varphi_{1j}^{\prime \prime }\left[\int_{0}^{L_{2}}\rho \varphi_{2k}dx+mX_{mk}\right]dx\\ -\int_{0}^{L_{2}}\varphi_{2s}Y_{mk}\left\{\rho \left[\varphi_{2j}^{\prime }+\left(x-L_{2}\right)\varphi_{2j}^{\prime \prime }\right]-m\varphi_{2j}^{\prime \prime }\right\}dx \end{array}\right\},\\ c_{jkr}^{s}=\frac{1}{M_{s}}\left\{\begin{array}{l} EI\int_{0}^{L_{1}}\varphi_{1s}{\left[\varphi_{1j}^{\prime }{\left(\varphi_{1k}^{\prime }\varphi_{1r}^{\prime \prime }\right)}^{¢}\right]}^{\prime }dx+EI\int_{0}^{L_{2}}\varphi_{2s}{\left[\varphi_{2j}^{\prime }{\left(\varphi_{2k}^{\prime }\varphi_{2r}^{\prime \prime }\right)}^{¢}\right]}^{\prime }dx\\ +k_{N}\left(\Theta_{1j}\Theta_{1k}\Theta_{1r}\Theta_{1s}+\Theta_{2j}\Theta_{2k}\Theta_{2r}\Theta_{2s}\right) \end{array}\right\},\\ d_{jkr}^{s}=\frac{1}{M_{s}}\left\{\begin{array}{l} \int_{0}^{L_{1}}\varphi_{1s}\left[\rho {\left(\varphi_{1j}^{\prime }\int_{L_{1}}^{x}\int_{0}^{x}\varphi_{1k}^{\prime }\varphi_{1r}^{\prime }dxdx\right)}^{\prime }-\left(\rho L_{2}+m\right)\varphi_{1j}^{\prime \prime }\int_{0}^{L_{1}}\varphi_{1k}^{\prime }\varphi_{1r}^{\prime }dx\right]dx\\ +\int_{0}^{L_{2}}\varphi_{2s}\left[\rho {\left(\varphi_{2j}^{\prime }\int_{L_{2}}^{x}\int_{0}^{x}\varphi_{2k}^{\prime }\varphi_{2r}^{\prime }dxdx\right)}^{\prime }-m\varphi_{2j}^{\prime \prime }\int_{0}^{L_{2}}\varphi_{2k}^{\prime }\varphi_{2r}^{\prime }dx\right]dx \end{array}\right\},\\ f_{s}(t)=\frac{1}{M_{s}}\left\{{\ddot{Y}}_{b}\left[\int_{0}^{L_{1}}\rho \varphi_{1s}dx+\left(\rho L_{2}+m\right)Y_{ms}\right]+{\ddot{X}}_{b}\left[\int_{0}^{L_{2}}\rho \varphi_{2s}dx+mX_{ms}\right]\right\}. \end{array}$$
